# Supplementary material for: Tomographic active optical trapping of arbitrarily shaped objects by exploiting 3D refractive index maps
Source: Nat Commun. 2017 May 22;8:15340. doi: 10.1038/ncomms15340 (PMC5458125; doi:10.1038/ncomms15340)
Supplement: Supplementary Information — Supplementary Figures, Supplementary Notes, Supplementary Table and Supplementary References [file ncomms15340-s1.pdf]

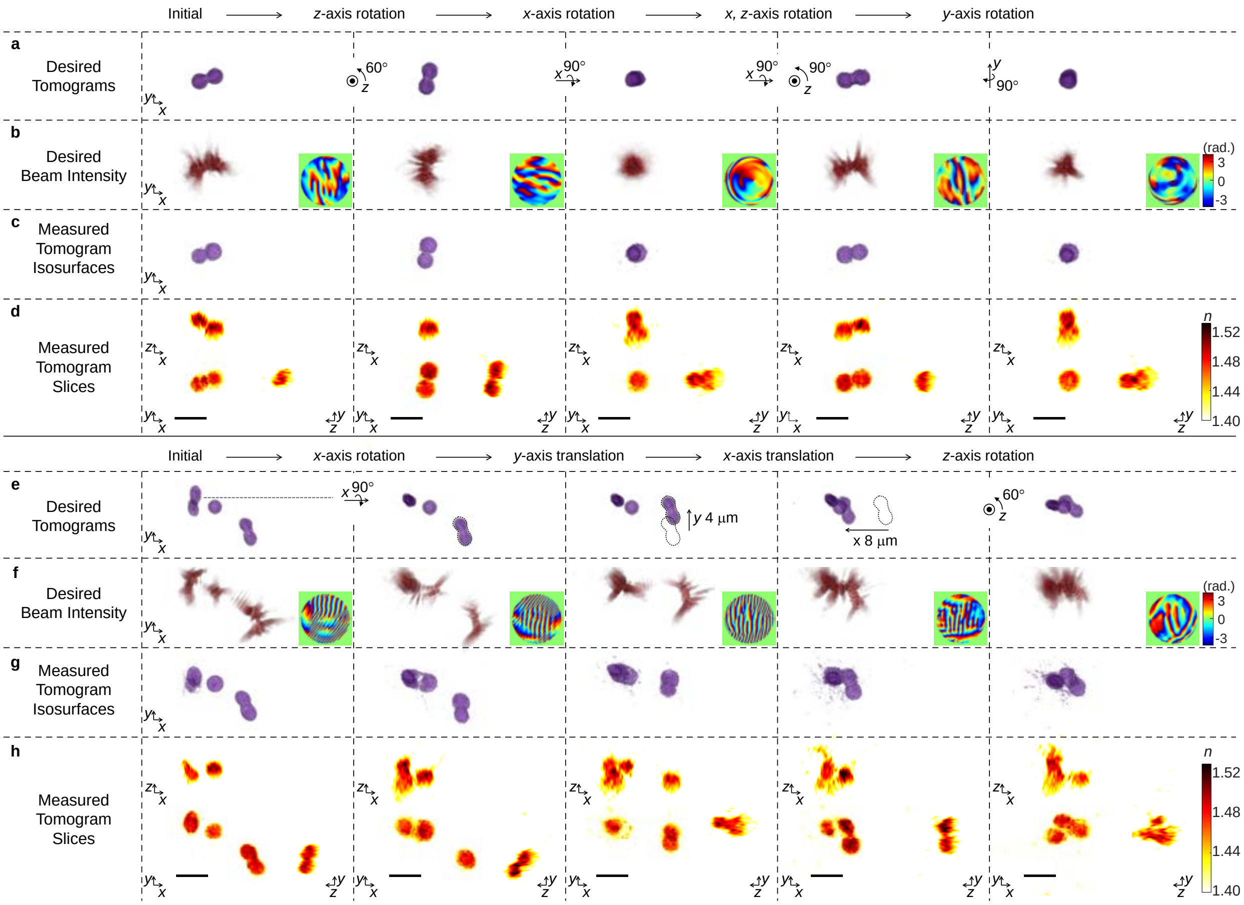

Supplementary Figure 1 | Controlling the orientation and assembly of colloidal PMMA particles. **a**-**d**, Time-lapse images of the controlled orientation of a PMMA dimer shown in Supplementary Movie 1. **e**-**h**, Time-lapse images of the assembly of three PMMA particles shown in Supplementary Movie 2. **a**, **e**, desired tomograms calculated by applying rotational, translational, and/or folding transformations to the reconstructed tomogram in the initial state. **b**, **f**, desired 3-D beam intensity generated by numerical propagation of the phase-only hologram in the insets of each column. The phase-only holograms were calculated by applying the 3-D Gerchberg-Saxton algorithm to the desired tomograms in each column in **a** and **e**, respectively. **c**, **g**, 3-D rendered isosurfaces of the tomograms of the PMMA particles trapped by the desired 3-D beam intensity in each column of **b** and **f**, respectively. **d**, **h**, the cross-sectional slice images of the measured tomograms in the *x*-*y*, *x*-*z*, and *y*-*z* plane. Scale bar indicates 5 μm.

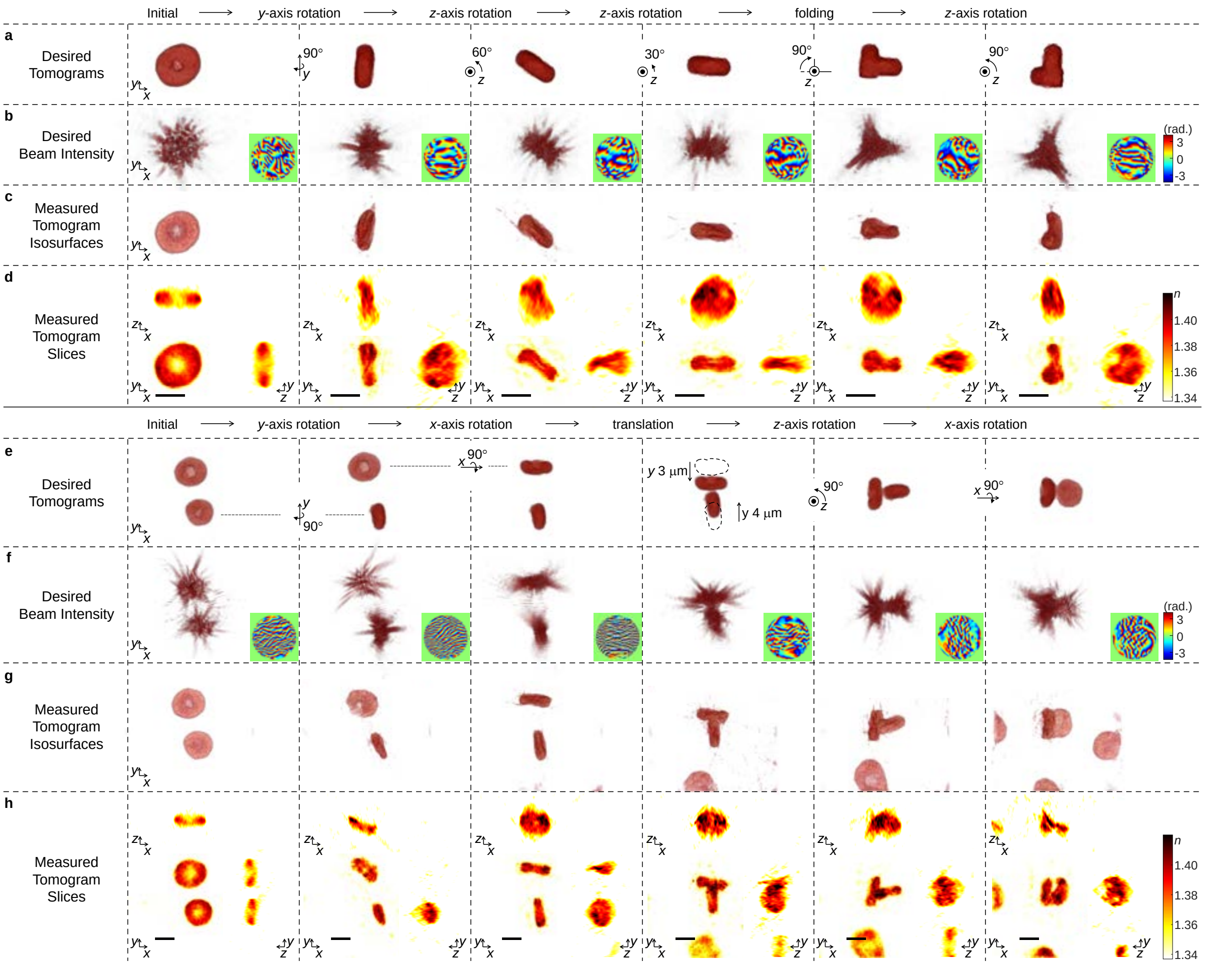

Supplementary Figure 2 | Controlling the orientation, shape and assembly of red blood cells. a-d, Time-lapse images of orientation control of individual red blood cells (RBCs) shown in Supplementary Movie 3. **e-h,** Time-lapse images of the assembly of two RBCs shown in Supplementary Movie 4. **a**, **e**, desired tomograms calculated by applying rotational, translational, and/or folding transformations to the reconstructed tomogram of the initial state. **b**, **f**, desired 3-D beam intensity generated by numerical propagation of the phase-only hologram in the insets of each column. The phase-only holograms were calculated by applying the 3-D Gerchberg-Saxton algorithm to the desired tomograms in each column in **a** and **e**, respectively. **c**, **g**, 3-D rendered isosurfaces of the tomograms of RBCs trapped by the desired 3-D beam intensity distribution in each column of **b** and **f**, respectively. **d**, **h**, the cross-sectional slice images of the measured tomograms in the *x*-*y*, *x*-*z*, and *y*-*z* planes. Scale bar indicates 5 μm.

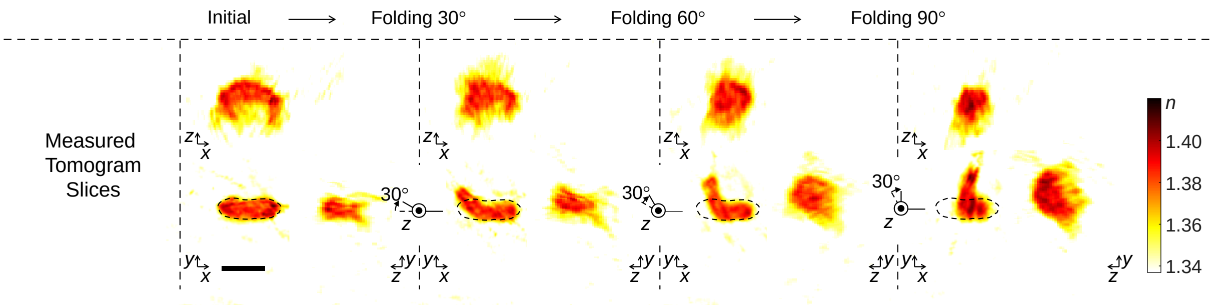

Supplementary Figure 3 | Folding of a red blood cell to an L-shaped cell. Time-lapse images of the intermediate steps for folding an individual red blood cell (RBC) to an L-shaped cell. The folding transformation was slightly modified from the folding transformation presented in the main text as a rotation transformation of one-half of the sample, whose rotation axis was shifted by 0.5 μm from the lower bisector of the sample. The 3-D RI distribution of the RBC folded by the modified folding transformation clearly shows the deformed portion of the RBC. Dashed lines indicate the shape of the RBC at the initial step. Scale bar indicates 5 μm. See also Supplementary Movie 6.

Supplementary Note 1 : Electromagnetic variational principle

The electromagnetic variational principle is analogous to the variational principle of quantum mechanics, which has been used to find approximations for ground energy states of given conditions. The electromagnetic variational principle deals with the minimization of the electromagnetic energy functional which differs from explicit equations for electric and magnetic energy stored in the electromagnetic fields. The electromagnetic variational principle has been conventionally used to find the lowest frequency mode of electric field patterns in a given arbitrary geometry of photonic crystals.

Here, we show that the electromagnetic variational principle can also be utilized for stable orientation control of arbitrarily shaped samples by finding electric field patterns aligning the samples in the ground state. Mathematical derivations for the electromagnetic variational principle were reprinted based on a reference[1].

In the absence of free charges and currents, Maxwell's equations for dielectric and nonmagnetic particles are:

$$\nabla \cdot \varepsilon \mathbf{E} = 0, \tag{1}$$

$$\nabla \cdot \mathbf{H} = 0, \tag{2}$$

$$\nabla \times \mathbf{E} = -\frac{\partial \mathbf{H}}{\partial t}, \tag{3}$$

$$\nabla \times \mathbf{H} = \varepsilon \frac{\partial \mathbf{E}}{\partial t}, \tag{4}$$

where $\mathbf{E}(\mathbf{r},t)$ and $\mathbf{H}(\mathbf{r},t)$ are the electric and magnetic field, respectively, and $\varepsilon(\mathbf{r})$ is the permittivity distribution of the non-dispersive particles.

When we only consider harmonic time-dependent field with a harmonic frequency ω,

$$\mathbf{E}(\mathbf{r},t) = \mathbf{E}(\mathbf{r})\exp(-i\omega t), \tag{5}$$

$$\mathbf{H}(\mathbf{r},t) = \mathbf{H}(\mathbf{r})\exp(-i\omega t). \tag{6}$$

Maxwell's equations become

$$\nabla \cdot \varepsilon \mathbf{E}(\mathbf{r}) = 0, \tag{7}$$

$$\nabla \cdot \mathbf{H}(\mathbf{r}) = 0, \tag{8}$$

$$\mathbf{H}(\mathbf{r}) = -\frac{i}{\omega}\nabla \times \mathbf{E}(\mathbf{r}), \tag{9}$$

$$\mathbf{E}(\mathbf{r}) = \frac{i}{\omega\varepsilon}\nabla \times \mathbf{H}(\mathbf{r}). \tag{10}$$

The magnetic field, $\mathbf{H}(\mathbf{r})$, can be rewritten by substituting the electric field in Supplementary Equation

(10) to Supplementary Equation (9) as:

$$\omega^2\mathbf{H} = \nabla\times\left(\varepsilon^{-1}(\mathbf{r})\nabla\times\mathbf{H}\right). \tag{11}$$

Supplementary Equation (11) can be interpreted as an eigenvalue problem,

$$\hat{H}\left|\mathbf{H}_\omega\right\rangle = \omega^2\left|\mathbf{H}_\omega\right\rangle, \tag{12}$$

where $\mathbf{H}_\omega$ is an eigenfunction of a Hermitian Maxwell Hamiltonian $\hat{H}$ as

$$\hat{H} = \nabla\times\left(\varepsilon^{-1}(\mathbf{r})\nabla\times\right). \tag{13}$$

The expectation value of the Hamiltonian $\hat{H}$ is

$$\left\langle\mathbf{H}_\omega\right|\hat{H}\left|\mathbf{H}_\omega\right\rangle = \int d^3\mathbf{r}\,\mathbf{H}^*(\mathbf{r})\cdot\nabla\times\left(\varepsilon^{-1}(\mathbf{r})\nabla\times\mathbf{H}(\mathbf{r})\right) = \int d^3\mathbf{r}\frac{1}{\varepsilon(\mathbf{r})}\left|\nabla\times\mathbf{H}(\mathbf{r})\right|^2, \tag{14}$$

and the last equation can be rewritten by replacing $\nabla\times\mathbf{H}(\mathbf{r})$ to Supplementary Equation (10)

$$\left\langle\mathbf{H}_\omega\right|\hat{H}\left|\mathbf{H}_\omega\right\rangle = \int d^3\mathbf{r}\frac{1}{\varepsilon(\mathbf{r})}\left|\nabla\times\mathbf{H}(\mathbf{r})\right|^2 = \int d^3\mathbf{r}\,\omega^2\varepsilon(\mathbf{r})\left|\mathbf{E}(\mathbf{r})\right|^2 = \omega^2\left\langle\mathbf{H}_\omega\middle|\mathbf{H}_\omega\right\rangle. \tag{15}$$

For that reason,

$$\left\langle\mathbf{H}_\omega\middle|\mathbf{H}_\omega\right\rangle = \int d^3\mathbf{r}\,\varepsilon(\mathbf{r})\left|\mathbf{E}(\mathbf{r})\right|^2. \tag{16}$$

Moreover, by substituting the magnetic field, $\mathbf{H}(\mathbf{r})$ in Supplementary Equation (14) with Supplementary Equation (9), Supplementary Equation (14) becomes

$$\left\langle\mathbf{H}_\omega\right|\hat{H}\left|\mathbf{H}_\omega\right\rangle = \int d^3\mathbf{r}\left[-\frac{i}{\omega}\nabla\times\mathbf{E}(\mathbf{r})\right]^*\cdot\nabla\times\left[\frac{1}{\varepsilon(\mathbf{r})}\left(-i\omega\varepsilon(\mathbf{r})\right)\mathbf{E}(\mathbf{r})\right] = \int d^3\mathbf{r}\left|\nabla\times\mathbf{E}(\mathbf{r})\right|^2 \tag{17}$$

The electromagnetic variational principle states that the electromagnetic energy functional

$$E_f(\psi) = \frac{\langle\psi|\hat{H}|\psi\rangle}{\langle\psi|\psi\rangle} \tag{18}$$

is minimized at the ground state $\left|\psi_g\right\rangle$ with energy E_g, and the value of the functional gives the energy of the ground eigenstate. By substituting Supplementary Equations (16-17) to Supplementary Equation (18), the electromagnetic energy functional becomes

$$E_f\left(\mathbf{H}_\omega\right)=\frac{\left\langle \mathbf{H}_\omega \middle| \hat{H} \middle| \mathbf{H}_\omega \right\rangle}{\left\langle \mathbf{H}_\omega \middle| \mathbf{H}_\omega \right\rangle}=\frac{\int d^3\mathbf{r}\left|\nabla\times\mathbf{E}(\mathbf{r})\right|^2}{\int d^3\mathbf{r}\,\varepsilon(\mathbf{r})\left|\mathbf{E}(\mathbf{r})\right|^2} \tag{19}$$

which is minimized when the electric field is concentrated in volumes of permittivity distribution in order to maximize the denominator of the quotient. For that reason, dielectric materials in optical fields tend to align with the high gradient of beam intensity in order to maximize the overlap volume between the materials and beam intensity.

Supplementary Note 2 : Feasibility of TOMOTRAP for the orientation control of a PMMA dimer

The feasibility of TOMOTRAP for the orientation control of a PMMA dimer was quantitatively analysed by calculating the 3-D cross-correlation values between the calculated tomogram (n_{mould}) of the desired orientation and the measured tomogram (n_{measured}). Time-lapse optical diffraction tomography was used to measure 20 tomograms of the PMMA dimer during the orientation change with the tomogram acquisition rate of 100 Hz. The 3-D cross-correlation values were calculated as the maximum value of the 3-D cross-correlation of two tomograms, $\langle n_{\mathrm{mould}}, n_{\mathrm{measured}}\rangle$, which were calculated by applying 3-D Fourier transforms as

$$\langle n_{\mathrm{mould}}, n_{\mathrm{measured}}\rangle = \mathcal{F}^{-1}\left[\left(\mathcal{F}\{n_{\mathrm{mould}}\}\right)^{*} \cdot \mathcal{F}\{n_{\mathrm{measured}}\}\right] \tag{20}$$

As shown in Supplementary Figure 4, the 3-D cross-correlation values were calculated as 0.92 ± 0.026 during the orientation changes and translation of the PMMA dimer. It clearly shows that the present method can control the orientation of arbitrarily shaped particles with high feasibility.

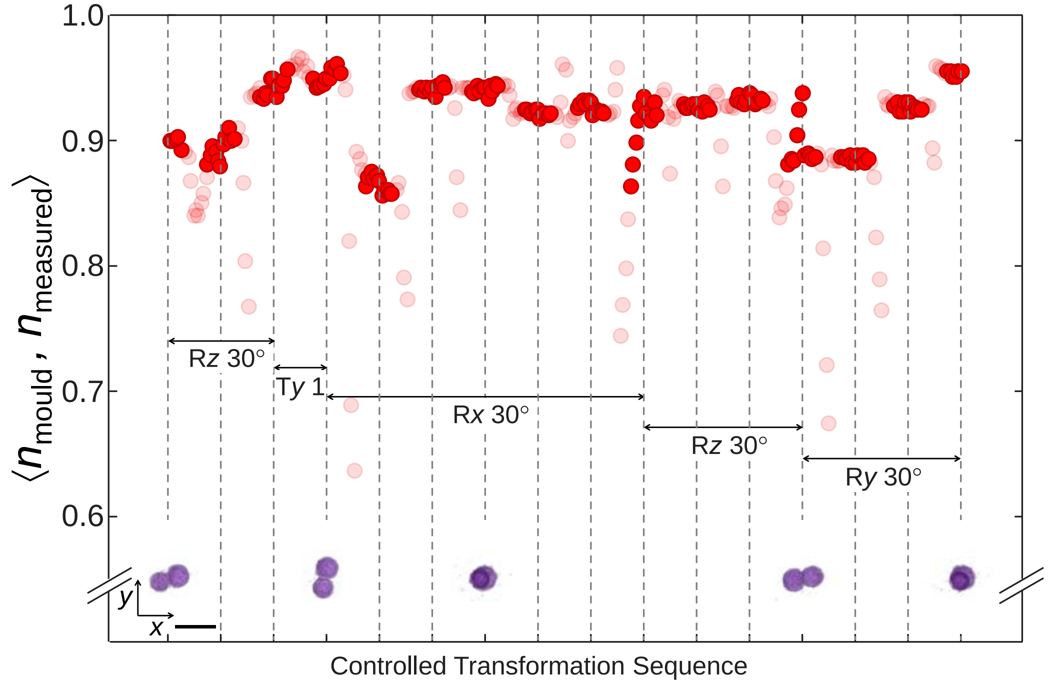

Supplementary Figure 4 | Feasibility of TOMOTRAP for the orientation control of a PMMA dimer. The 3-D cross-correlation values between the desired tomograms and the measured tomograms during the orientation control of a PMMA dimer. The rotation or translation transformation were applied at every dashed line, which are indicated as Rα ##° and Tβ *, respectively. Rα ##° symbolizes the transformation for rotating the PMMA dimer ##° with respect to the α-axis, and Tβ * symbolizes the transformation for translating the PMMA dimer by * μm along the β-axis. For instance, rotation transformation for rotating the PMMA dimer 30° with respect to the x-axis (Rx 30°) was applied 6 times. Solid red circles are the 3-D cross-correlation values when the PMMA dimer is trapped steadily in the 3-D beam intensity of desired tomograms, while shaded circles indicate intermediate states during the orientation change in response to applying the next translation and/or rotation transformation. The representative 3-D rendered isosurfaces of the measured tomograms are presented in the inset of the corresponding translation/rotation transformation. Scale bar indicates 5 μm.

Supplementary Note 3 : Translational and rotational trap stiffness of TOMOTRAP

We quantitatively measured translational and rotational trap stiffness for trapping a PMMA dimer in various desired orientations. For systematic investigation of the translational and rotational trap stiffness of TOMOTRAP, we controlled the orientation of the PMMA dimer with the polar angle, θ', from 0 to 90° with the increment of 30° and the azimuthal angle, φ', from -90 to 0° with the increment of 30°, as indicated in Supplementary Figures 5a-b. Moreover, we trapped the same dimer with a Gaussian beam in order to compare the trap stiffness trapped by the Gaussian beam and TOMOTRAP with the desired orientation of $\theta' = 0°$.

In order to measure the translational and rotational trap stiffness for trapping the PMMA dimer, we measured 150 time-lapse tomographic images of the dimer with the tomogram acquisition rate of 90 Hz during trapping with the desired orientation. From each 3-D refractive index (RI) distribution, the centroids and principal axes of the PMMA dimer were extracted, and we transformed measured position and angle displacement of the dimer in the laboratory frame (x_{lab}, y_{lab}, z_{lab}) to the probe frame to extract generalized coordinates $\mathbf{q} = (\mathbf{r}, \theta, \varphi)$ of the sample away from the equilibrium position and orientation, as indicated in Supplementary Figure 5a. Since the PMMA dimer has a rotational symmetry with the axis of symmetry along the z-axis in the probe frame, we can track five degrees of freedom consisting of three translational degrees of freedom ($\mathbf{r} = x, y, z$) and two rotational degrees of freedom (θ, φ).

From the time evolution of generalized coordinates of PMMA dimers trapped by TOMOTRAP, the translational and rotational trap stiffness were calculated using the equipartition theorem as[2]:

$$\frac{1}{2}k_B T I = \frac{1}{2}K\left\langle \mathbf{q}\otimes\mathbf{q}\right\rangle, \tag{21}$$

where I is an identity matrix, K is a trap stiffness matrix which diagonal components provide the trap stiffness of TOMOTRAP along each degree of freedom, and $\langle\mathbf{q}\otimes\mathbf{q}\rangle$ is a covariance matrix of the generalized coordinates of the sample.

The calculated translational stiffness along the x-, y-, and z-axis of the probe frame of the PMMA dimer is shown in Supplementary Figure 5c. The translational stiffness in all three directions increases as θ' decreases, which implies that the PMMA dimer is trapped in a more stable manner when the dimer is more aligned in the optical axis. Nonetheless, the translational stiffness of the PMMA dimer at $\theta' = 90°$ in each axis shows that the PMMA dimer in face-on orientation can be trapped stably with the translational stiffness of tens of pN μm^{-1}. Moreover, the translational stiffness of the PMMA dimer in edge-on orientation ($\theta' = 0°$) trapped by TOMOTRAP is comparable to a Gaussian trap.

The rotational trap stiffness of the trapped PMMA dimer increases as θ' decreases (Supplementary Figure 5d), which is a similar tendency to the translational trap stiffness. Interestingly, the magnitude of the rotational trap stiffness for trapping the PMMA dimer along the x- and y-axis, k_φ and k_θ respectively, with the orientation of the same θ' seems to alternate as φ' changes, especially when θ' is larger than 60°. When the dimer is aligned along the y_{lab}-axis as $\varphi' = -90°$, k_θ dominates k_φ. As φ' increases, k_θ decreases while k_φ increases, and eventually k_φ dominates k_θ when the dimer is aligned along the x_{lab}-axis as $\varphi' = 0°$. We presumed that this tendency is related to the linearly polarized trapping beam, which requires further investigations.

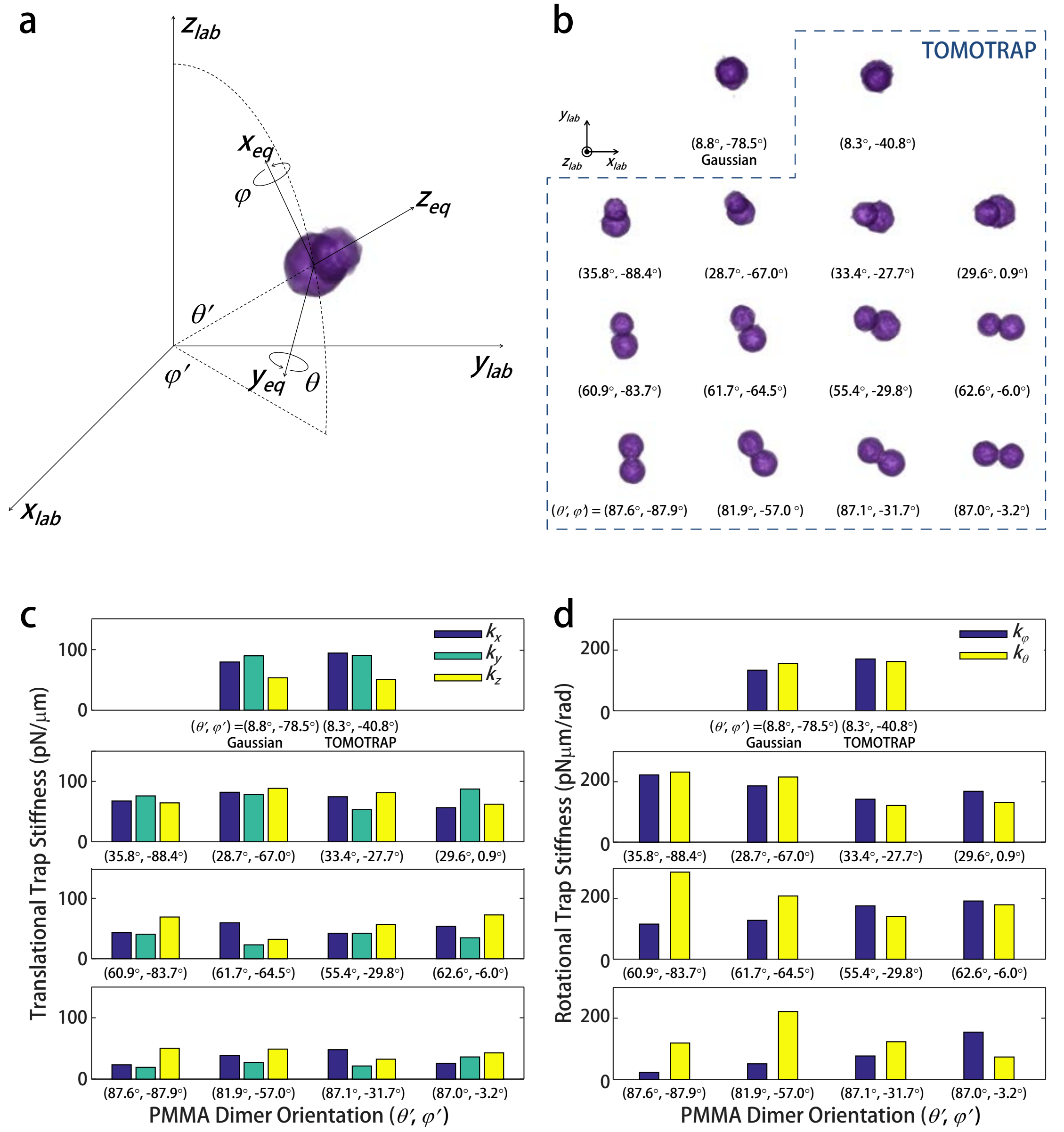

Supplementary Figure 5 | The translational and rotational trap stiffness for trapping a PMMA dimer by TOMOTRAP. a, the coordinate system describing the laboratory frame and the probe frame of the trapped PMMA dimer. The origin of the probe frame is located at the averaged centroid position of the PMMA dimer, and the x_{eq}- and y_{eq}- and z_{eq}-axis is parallel to the principal axis of the averaged 3-D refractive index (RI) distribution of the PMMA dimer. **b**, The 3-D RI isosurfaces of the PMMA dimer trapped by a Gaussian beam and TOMOTRAP in various orientations (θ', φ'). We controlled the orientation of the PMMA dimer with the polar angle, θ', from 0 to 90° with the increment of 30° and the azimuthal angle, φ', from -90 to 0° with the increment of 30°. Each row presents 3-D RI isosurfaces of the PMMA dimer with the same polar angle, θ'. **c** and **d**, The translational and rotational trap stiffness of the Gaussian trap and TOMOTRAP in various orientations of the PMMA dimer. Each row in **c** and **d** presents the translational and rotational trap stiffness of the PMMA dimer with the same polar angle, θ'.

Supplementary Note 4 : Trap stiffness of TOMOTRAP for trapping spherical particles with various diameters

We compared the trap stiffness for trapping the different sized spherical particles with a regular single focused (Gaussian) beam and the 3-D Gerchberg-Saxton (GS) shaped beam generated by the proposed method in the same laser power (80 mW at the sample plane). We used silica (n = 1.4607 at λ = 532 nm) spheres with diameters of 2 μm and 5 μm, and PMMA (n = 1.4937) spheres with diameters of 3, 4, and 8 μm immersed in 45% sucrose solution (n = 1.41) in order to reduce the refractive index contrast between samples and surrounding media. A Gaussian beam and the 3-D GS shaped beam trapped the same bead in turn, and a quadrant photo diode (QPD, PDQ80A, Thorlabs Inc.) measured temporal position fluctuations of the bead for 8 seconds with a sampling rate of 80 kHz. The trap stiffness along the *x*- and *y*-axis was calculated as the corner frequency of the Lorentzian curve fitting the power spectrum of the measured position fluctuations[3] (Supplementary Figure 6).

As shown in Supplementary Figure 7 and Supplementary Table 1, the enhancement of the corner frequency is not significant when trapping spherical particles with diameters of 2 μm and 3 μm, and we believe that it results from the small size of the spherical particles so that the difference between the 3-D GS shaped beam and a Gaussian trap is minimal. For larger sized particles, however, the trap stiffness is enhanced in both *x*- and *y*-axis when trapped by the 3-D GS shaped beam which shape corresponds to reconstructed 3-D RI distribution of the sphere. The enhancement factor of the trap stiffness increases as the size of the bead increases, as the trap stiffness for trapping a silica bead with a diameter of 5 μm shows 1.8 – 1.9 fold enhancement in TOMOTRAP. The results show that TOMOTRAP can enhance the trap stiffness of optical tweezers by considering the shape of trapped samples. The enhancement factor for trap stiffness drops for trapping a PMMA bead with a diameter of 8 μm, which may originate from the loosely focused laser beam for trapping a large particle.

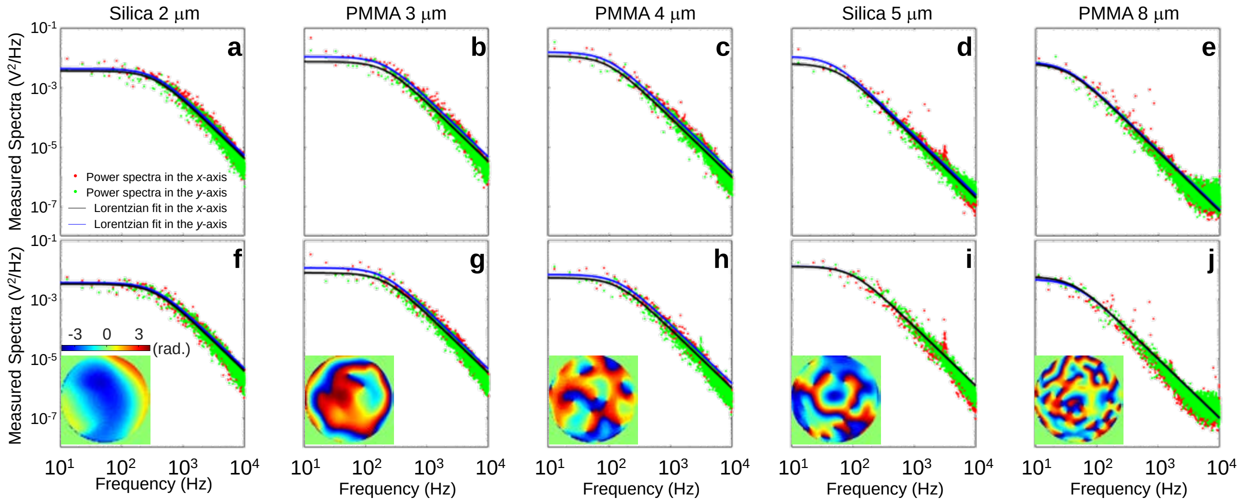

Supplementary Figure 6 | Measured power spectra for trapping spherical particles. Measured power spectra for trapped spherical particles with the different diameters: 2, 3, 4, 5, and 8 μm, as indicated for each column. **a**-**e**, measured power spectra for spherical particles trapped by a regular focused (Gaussian) beam. **f**-**j**, measured power spectra for spherical particles trapped by the 3-D Gerchberg-Saxton (GS) shaped beams generated by the proposed method. Insets at each column indicate the phase-only hologram which generates the 3-D GS shaped beam for trapping the spherical particles with different diameters. Solid red and green points indicate the measured power spectra in the *x*- and *y*-axis, respectively, and black and blue lines indicate the Lorentzian fit of the measured power spectra in the *x*- and *y*-axis, respectively.

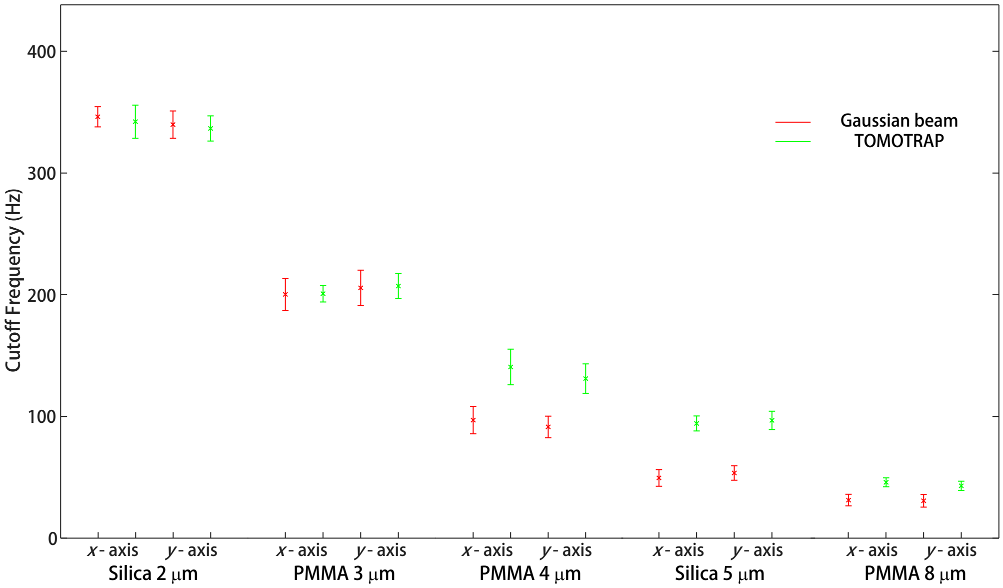

Supplementary Figure 7 | The corner frequency of the measured power spectra along the *x*- and *y*-axis for spherical particles with different diameters which were trapped by a Gaussian beam (red) and a 3-D beam intensity shaped by TOMOTRAP (green). Error bars correspond to the standard deviation of 20 corner frequency frequencies for each data point.

Supplementary Table 1 | The corner frequency of the measured power spectra along the *x*- and *y*-axis for spherical particles with different diameters trapped by a Gaussian beam and a 3-D beam intensity shaped by TOMOTRAP.

	Corner frequency in the *x* - axis			Corner frequency in the *y* - axis		
	Gaussian (Hz)	TOMOTRAP (Hz)	Enhancement	Gaussian (Hz)	TOMOTRAP (Hz)	Enhancement
Silica 2 μm	347.0 ± 8.3	343.1 ± 13.6	0.99	340.6 ± 11.2	337.5 ± 10.3	0.99
PMMA 3 μm	200.3 ± 13.1	200.9 ± 6.8	1.00	205.7 ± 14.6	207.2 ± 10.4	1.01
PMMA 4 μm	97.0 ± 11.3	140.7 ± 14.7	1.45	91.4 ± 8.9	131.1 ± 12.1	1.43
Silica 5 μm	49.5 ± 6.8	94.3 ± 6.2	1.90	53.6 ± 6.0	96.8 ± 7.5	1.81
PMMA 8 μm	31.0 ± 4.8	45.6 ± 3.6	1.47	30.5 ± 5.2	42.8 ± 3.8	1.4

Supplementary References

1. Joannopoulos, J. D., Johnson S. G., Winn J. N. & Meade R. D. *Photonic crystals: molding the flow of light*, 2nd edn. (Princeton University Press, 2008).

2. Phillips, D. B. *et al.* An optically actuated surface scanning probe. *Opt. Express* **20**, 29679-29693 (2012).

3. Berg-Sørensen, K. & Flyvbjerg H. Power spectrum analysis for optical tweezers. *Rev. Sci. Instrum.* **75**, 594-612 (2004).
